# Charged Particle Irradiation for Pancreatic Cancer: A Systematic Review of *In Vitro* Studies

**DOI:** 10.3389/fonc.2021.775597

**Published:** 2022-01-04

**Authors:** Dandan Wang, Ruifeng Liu, Qiuning Zhang, Hongtao Luo, Junru Chen, Meng Dong, Yuhang Wang, Yuhong Ou, Zhiqiang Liu, Shilong Sun, Kehu Yang, Jinhui Tian, Zheng Li, Xiaohu Wang

**Affiliations:** ^1^ Institute of Modern Physics, Chinese Academy of Sciences, Lanzhou, China; ^2^ The First School of Clinical Medicine, Lanzhou University, Lanzhou, China; ^3^ Department of Postgraduate, University of Chinese Academy of Sciences, Beijing, China; ^4^ Heavy Ion Therapy Center, Lanzhou Heavy Ions Hospital, Lanzhou, China; ^5^ Evidence-Based Medicine Center, School of Basic Medical Sciences, Lanzhou University, Lanzhou, China

**Keywords:** pancreatic cancer, particle radiation, clonogenic survival, DDR, migration, invasion, systematic review

## Abstract

**Purpose:**

Given the higher precision accompanied by optimized sparing of normal tissue, charged particle therapy was thought of as a promising treatment for pancreatic cancer. However, systematic preclinical studies were scarce. We aimed to investigate the radiobiological effects of charged particle irradiation on pancreatic cancer cell lines.

**Methods:**

A systematic literature search was performed in EMBASE (OVID), Medline (OVID), and Web of Science databases. Included studies were *in vitro* English publications that reported the radiobiological effects of charged particle irradiation on pancreatic cancer cells.

**Results:**

Thirteen carbon ion irradiation and seven proton irradiation *in vitro* studies were included finally. Relative biological effectiveness (RBE) values of carbon ion irradiation and proton irradiation in different human pancreatic cancer cell lines ranged from 1.29 to 4.5, and 0.6 to 2.1, respectively. The mean of the surviving fraction of 2 Gy (SF2) of carbon ion, proton, and photon irradiation was 0.18 ± 0.11, 0.48 ± 0.11, and 0.57 ± 0.13, respectively. Carbon ion irradiation induced more G2/M arrest and a longer-lasting expression of γH2AX than photon irradiation. Combination therapies enhanced the therapeutic effects of pancreatic cell lines with a mean standard enhancement ratio (SER) of 1.66 ± 0.63 for carbon ion irradiation, 1.55 ± 0.27 for proton irradiation, and 1.52 ± 0.30 for photon irradiation. Carbon ion irradiation was more effective in suppressing the migration and invasion than photon irradiation, except for the PANC-1 cells.

**Conclusions:**

Current *in vitro* evidence demonstrates that, compared with photon irradiation, carbon ion irradiation offers superior radiobiological effects in the treatment of pancreatic cancer. Mechanistically, high-LET irradiation may induce complex DNA damage and ultimately promote genomic instability and cell death. Both carbon ion irradiation and proton irradiation confer similar sensitization effects in comparison with photon irradiation when combined with chemotherapy or targeted therapy.

## Introduction

Pancreatic cancer is one of the most aggressive cancers—of which pancreatic ductal adenocarcinoma (PDAC) is the most frequent type, accounting for 85% of all cases—and is associated with the highest mortality rate (1). The efficacy of available treatments is limited; only approximately 15–20% of all patients can be treated with an R0 resection at the time of diagnosis (2). However, even after resection, a high proportion of patients die from local recurrence and/or distant metastasis (3). New therapies or therapeutic combinations are therefore required to improve outcomes from this cancer type, which are currently very poor. Radiation therapy combined with chemotherapy is an important treatment modality; however, pancreatic cancer is extremely hypoxic, resulting in epithelial to mesenchymal transition and resistance to low linear energy transfer (LET) radiation (4). It has been reported that the 1-year local control rates associated with conventional chemoradiotherapy (CRT) in people with PDAC is just 40–60% (5). Moreover, in conventional photon irradiation, the dose cannot be escalated owing to the risk of toxicity in the surrounding radiosensitive organs, such as the small bowel, liver, and kidneys.

The prevailing use of highly sophisticated, photon-based external beam radiation techniques including stereotactic body radiation therapy (SBRT), intensity-modulated radiotherapy (IMRT) offers more conformal dose distributions and have shed light to people with pancreatic cancer. A meta-analysis estimated that in locally advanced pancreatic cancer (LAPC), the 2-year overall survival (OS) is 26.9% with SBRT and just 14.7% for CRT (6).Charged particle (carbon ion and proton) represents an emerging technological advance in oncology and yielded quite encouraging outcomes. There are articles demonstrated the 2-year OS of LAPC for carbon ion radiotherapy (CIRT) with concurrent gemcitabine (GEM) was approximately 50%, and the 1-year OS of proton radiotherapy (PRT) concurrent chemotherapy was approximately 76%. In addition, CIRT and PRT can significantly reduce toxicity in those who receive these therapies (7–10).

Compared with conventional photon radiation, proton offers the potential physical advantage of improved dose localization offered by a spread-out Bragg peak (SOBP). It can deliver equivalent doses to targets as photons would while sparing integral dose to organs at risk (OARs), which could potentially reduce toxicity (11). Compared with proton, carbon ion offers comparable physical characteristics (12), but has substantially different biological properties. Carbon ion is associated with an enhanced relative biological effectiveness (RBE) due to higher LET, which theoretically induce more direct DNA damage, and double-strand breaks (DSBs). Carbon ion irradiation is also more effective against hypoxic radioresistant tumors, due to its low oxygen enhancement ratio. These characteristics make charged particle therapy a very promising cancer treatment option, however the exact radiobiological responses to charged particle irradiation have not been fully elucidated. It is unclear what modifications charged particle irradiation induces to the DNA and to signal transduction events. Moreover, the effects of combining carbon ion/proton irradiation and drugs with various mechanisms of action may differ from those of photon irradiation.

Several *in vitro* publications have explored the radiobiological effects of charged particle irradiation for pancreatic cancer. However, these studies differed in the cellular origins, charged particle type, radiation dose, dose rate, and therapeutic combinations (13–15). Therefore, further investigation is needed to combine and assess the current knowledge of this topic offered by *in vitro* studies. This study is a systematic review (SR) of published *in vitro* studies on the topic of carbon ion/proton irradiation for pancreatic cancer. It includes a comprehensive analysis of the cellular and molecular effects of charged particle irradiation on pancreatic cancer cell lines to gain insights into the mechanisms of particle therapy alone or as part of combination therapies used to treat pancreatic cancer.

## Materials and Methods

This SR was developed according to the Preferred Reporting Items for Systematic Reviews and Meta-Analyses (PRISMA) guidelines (16).

### Search Strategy

We used database-specific subject headings and free-text terms describing the populations or interventions to search Embase (OVID), Medline (OVID), and Web of Science databases (dates of inception to August 27, 2021). In addition, these terms were also used to access any unpublished material using Google search. Finally, the references of included studies were checked manually to identify any initially omitted publications. The full search strategy is described in the **Supplementary Materials**.

### Study Selection

After independently screening the titles and abstracts, two trained reviewers intensively read the full text to determine the final inclusion. Disagreements were discussed with the participation of a third reviewer.

The inclusion criteria for this research were:

1. *In vitro* studies of pancreatic cancer cell lines irradiated by carbon ion/proton.
2. Reported at least one of the following outcomes:
   2.1. Cell clonogenic survival.
   2.2. DNA damage response (DDR): cell cycle checkpoints, DNA repair, and apoptosis.
   2.3. Migration or invasion.
   2.4. The standard enhancement ratio (SER) evaluating the therapeutic effects of combination therapy. For studies that failed to report SER, we extracted data from published plots using Web Plot Digitizer and then determined the SER by calculating the ratio of doses in treated and control groups for a given isoeffect (surviving fraction (SF)=0.1) (17).
3. Articles published in English.

The exclusion criteria were:

1. Artificially modified cells lines.
2. No corresponding outcome was reported.
3. Clinical and animal studies, case reports, reviews, commentary, expert opinion, conference abstracts, correspondence.
4. Pilot studies and research projects.
5. Full-text articles were not available.

### Data Extraction

Two trained reviewers independently extracted data using a pre-established data extraction form. The following details were collected: name of the first author and the publication year, country, or region, type of particle irradiation, cell type (origin), the carbon ion/proton irradiation schedule (initial energy, average LET, SOBP, single doses, dose rate, and dose group), combination therapy, type of photon radiation. The extracted data were confirmed by a third reviewer.

### Assessment of Risk of Bias

For *in vitro* studies, no standard risk of bias assessment instrument exists now, so we produced these criteria ourselves, detailed in the supplementary materials (**Table S1**). These criteria evaluated the risk of bias induced by selection, performance, detection, attrition, cell-related, and other bias. The risk of bias was categorized into “Low”, “Moderate”, or “High”. When a study lacked sufficient details to evaluate, the risk of bias was categorized as “Risk Unknown”. In duplicate, two reviewers assessed the risk of bias. Disagreements were solved by the participation of a third reviewer.

### Statistical Analysis

In general, we used descriptive statistics to summarize baseline variables. The continuous data were presented as mean with standard deviation (SD) or median with interquartile ranges (IQR). All analyses were performed using R 4.0.3. A value of P < 0.05 was thought statistically significant.

## Results

### Search Results

**Figure 1:**
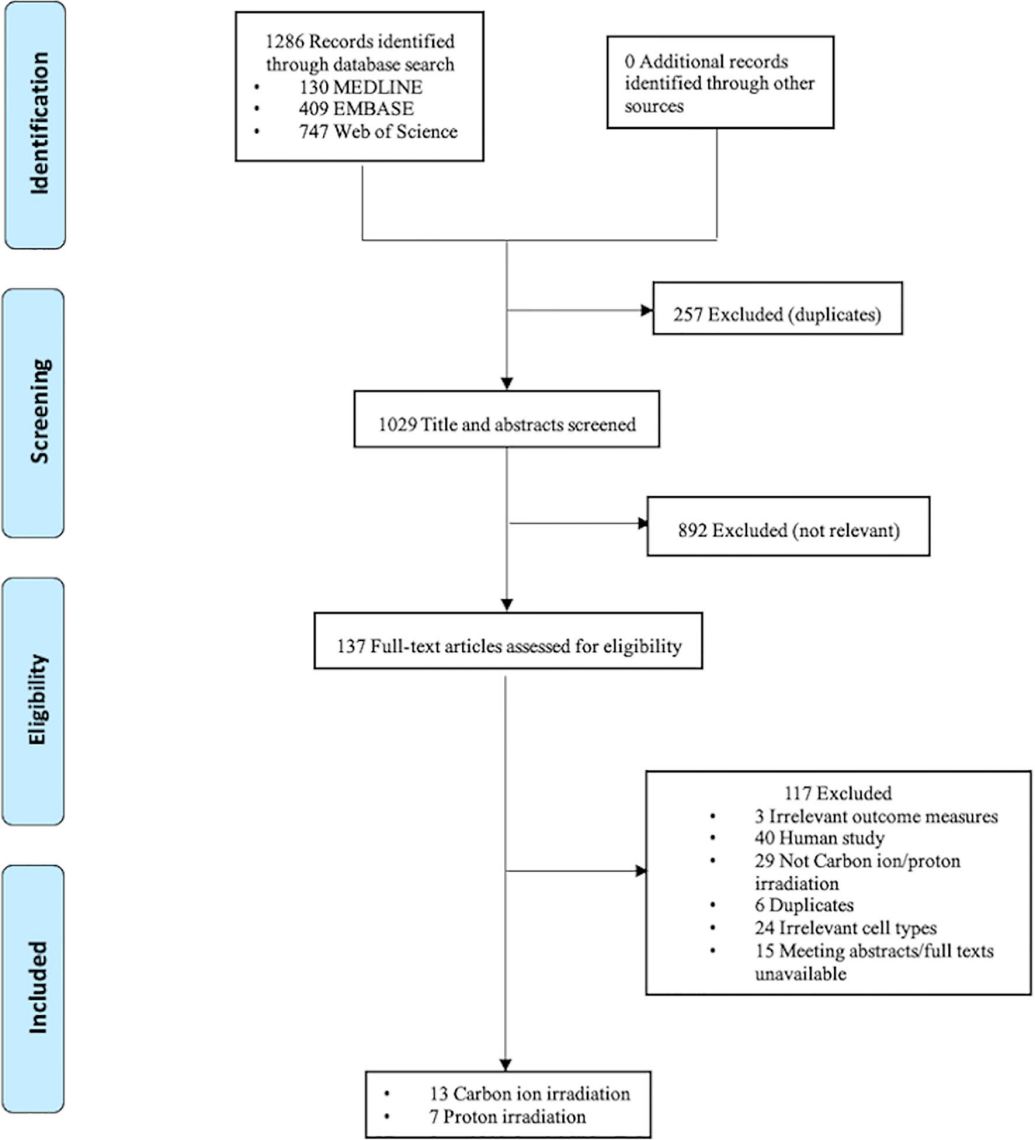
PRISMA flow diagram.

The electronic search resulted in 1029 unique citations. After reference and full-text screening, a total of 20, including 13 on carbon ion irradiation and seven on proton irradiation, met the eligibility criteria. The screening and selection processes are presented in **Figure 1**.

### Study Characteristics

The characteristics of included (13–15, 18–34) studies are summarized in **Table 1**. Briefly, 13 carbon ion irradiation and seven proton irradiation studies were included in our final SR; these studies were published between 2004 and 2021.They were conducted in 7 different countries: Japan, Germany, Belgium, Italy, Korea, the United States of America, and Lithuania. Of these studies, almost all used human pancreatic cancer cell lines, such as AsPC-1, BxPc-3, MIA PaCa-2, PANC-1, and PK45, only one (18) used transgenic mice cell lines (PDA30364/OVA). The studies varied in the initial energy, averaged LET, SOBP, radiation doses, and dose rates used. Two studies (14, 26) explored the effects of carbon ion irradiation on human pancreatic cancer stem-like cells (CSCs), using CD44^+^CD24^+^ESA^+^ and CD133^+^ as markers for their identification (35, 36). Carbon ion/proton irradiation combined with GEM was reported in three studies (20, 26, 29), poly (ADP-ribose) polymerase (PARP) inhibitor (PARPi) in three studies (24, 31, 33), B02 (a RAD51 inhibitor) in one study (33) and camptothecin (CPT) in another study (27). In addition, one study (19) combined carbon ion irradiation, magnetic nanoparticles (MNPs), and hyperthermia (Hyp) as a new treatment combination for pancreatic cancer. In total, 13 control groups were treated using X-rays, one with γ-rays, and the remaining six control groups were not irradiated. Eleven studies reported RBE values. The study by Hirai and colleagues (24) did not report the RBE values, but they calculated the isoeffective doses of γ-ray, LET 13 carbon ion, and LET 70 carbon ion irradiations that resulted 10% cell survival for their follow-up analyses.

**Table 1:** Overview of the included *in vitro* studies.

Author (year)	Country	Charged particle	Cell type	Initial energy	Average LET	SOBP	Dose rate	Dose group	Combination therapy	Control
Oonishi, K. 2012 (14)	Japan	carbon ion	MIA PaCa-2, BxPc-3^*^	290Mev/n	50KeV/μm	6cm	–	1, 2, 3Gy	–	X ray
Hartmann, L. 2020 (18)	Germany	carbon ion	PDA30364/OVA^†^	–	103KeV/μm	8mm	–	0.1, 0.4, 1.0, 3.1Gy	–	X ray
Sai, S. 2015 (26)	Japan	carbon ion	PK45, PANC1, MIA PaCa-2, BxPc-3^*^	290MeV/n	50keV/μm	6cm	–	1Gy	GEM	X ray
Fujita, M. 2015 (13)	Japan	carbon ion	MIA PaCa-2, AsPC-1, BxPc-3, PANC-1^*^	290MeV/n	80keV/μm	NR	1Gy/min	0.5, 1, 2, 4Gy	–	X ray
Hirai, T. 2012 (24)	Japan	carbon ion	MIA PaCa-2^*^	290MeV/n	13 and 70 keV/μm	NR	1.2Gy/min	–	Ola	γ ray
Schlaich, F. 2013 (27)	Germany	carbon ion	PANC-1^*^	–	103keV/μm	NR	0.5Gy/min	0.5, 1, 2, 3Gy	CPT	X ray
El Shafie, R. A. 2013 (20)	Germany	carbon ion	AsPC-1, BxPc-3, PANC-1^*^	–	103keV/μm	NR	–	0.125, 0.5, 1, 2, 3Gy	GEM	X ray
Matsui, Y. 2004 (25)	Japan	carbon ion	MIA PaCa-2, SUIT2, BxPc-3^*^	–	13/50/80 keV/μm	6cm	0.85Gy/min	–	–	X ray
Brero, F. 2020 (19)	Italy	carbon ion	BxPC3^*^	246-312 MeV/u	45keV/μm	6cm	–	0-2Gy	–	X ray
Fujita, M. 2012 (23)	Japan	carbon ion	MIA PaCa-2, AsPC-1, BxPc-3, PANC-1^*^	290MeV/u	80 keV/μm	NR	1Gy/min	0, 0.5, 1, 2, 4Gy	–	X ray
Facoetti, A. 2018 (21)	Italy	carbon ion	AsPC-1^*^	246-312 MeV/u		NR	1.39Gy/min/cm2		–	X ray
Fujita, M. 2014 (22)	Japan	carbon ion	MIA PaCa-2, PANC-1^*^	290MeV/u	80 keV/μm	NR	1Gy/min	0.5, 1, 2, 4Gy	–	No IR
Lee, S. H. 2021 (34)	Japan	carbon ion	MIA PaCa-2^*^	313.2/288.0/261.5 MeV/u	12.5/26.4/48.8/87.9 keV/μm	6cm	–	0-5Gy	–	No IR
Lee, Min-Gu 2019 (32)	Korea	proton	Capan-1, PANC-1^*^	100MeV	–	6cm	–	2, 4, 8 or 16Gy	–	No IR
Fujinaga, H. 2019 (28)	Japan	proton	MIA PaCa-2^*^	200MeV	–	NR	–	8Gy	–	X ray
Wera, Anne-Catherine 2019 (33)	Belgium	proton	KP4, PANC-1*	1.3MeV	25keV/μm	NR	2Gy/min	0.5, 1 Gy	Ola B02	X ray
Liubavičiūtė, A. 2015 (30)	Lithuania	proton	MIA PaCa-2^*^	20nA and 1.6-MeV	–	NR	–	1.6Gy	–	No IR
Galloway, N. R. 2009 (29)	US	proton	PANC-1, MIA PaCa-2^*^	250MeV	–	NR	2.5/5/10/15Gy/h	0-15Gy	GEM	No IR
Görte, J. 2020 (15)	Germany	proton	BxPC3, PANC-1, MIA PaCa-2, Patu8902^*^	150MeV	3.7 keV/μm	26.3mm	–	2, 4, 6Gy	–	X ray
Hirai, T. 2016 (31)	Japan	proton	MIA PaCa-2^*^	160MeV	4.3KeV/μm	NR	2.5Gy/min	–	Ola	No IR

CPT, camptothecin; GEM, gemcitabine; NR, not reported; Ola, Olaparib; IR, irradiation.

*human pancreatic cancer cell lines; †pancreatic adenocarcinoma cell line from transgenic mice.

### Risk of Bias

**Figure 2:**
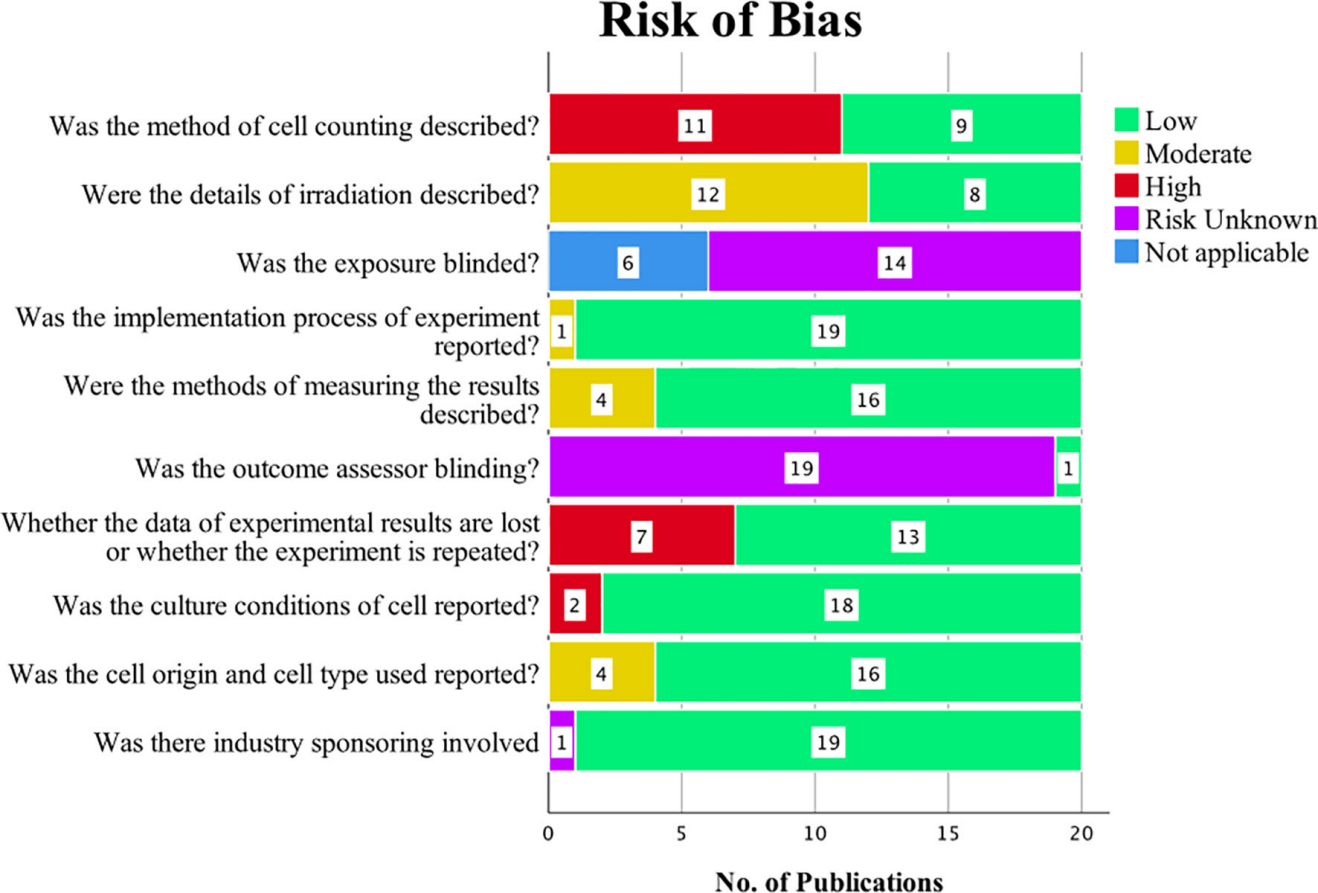
Results of the risk of bias assessment.

The outcomes of the risk of bias evaluation are shown in **Figure 2**. Among the 20 *in vitro* studies included in our SR, eight studies (13, 15, 19, 20, 22, 23, 27, 30) provided adequate information on irradiation. Eleven of the studies failed to describe cell counting methods. Seven studies (14, 15, 19, 23, 28, 29, 34) did not report whether the experiments were repeated, resulting in attrition bias. None of the publications stated whether the selection of treatment was blinded, and only one paper (21) reported blinding the outcomes assessors to minimize detection bias. All papers except one (28) described the implementation process. Four publications (19, 25, 28, 33) only partly described how results were measured; the remaining 16 described these methods in detail. One study (23) did not mention the conditions in which cells were cultured, and four studies (18, 31, 33, 34) partly reported the cell origin and cell type. Only one study (25) did not clear whether there was industry sponsoring; the rest were reported to have no connections with or employment at a company.

### RBE Values

RBE is defined as the ratio of the amount of dose from test radiation required to generate the same biological endpoint (usually cell-killing) relative to reference radiation (usually 250 kVp X-rays or Co-60 γ-rays) (37).

**Figure 3:**
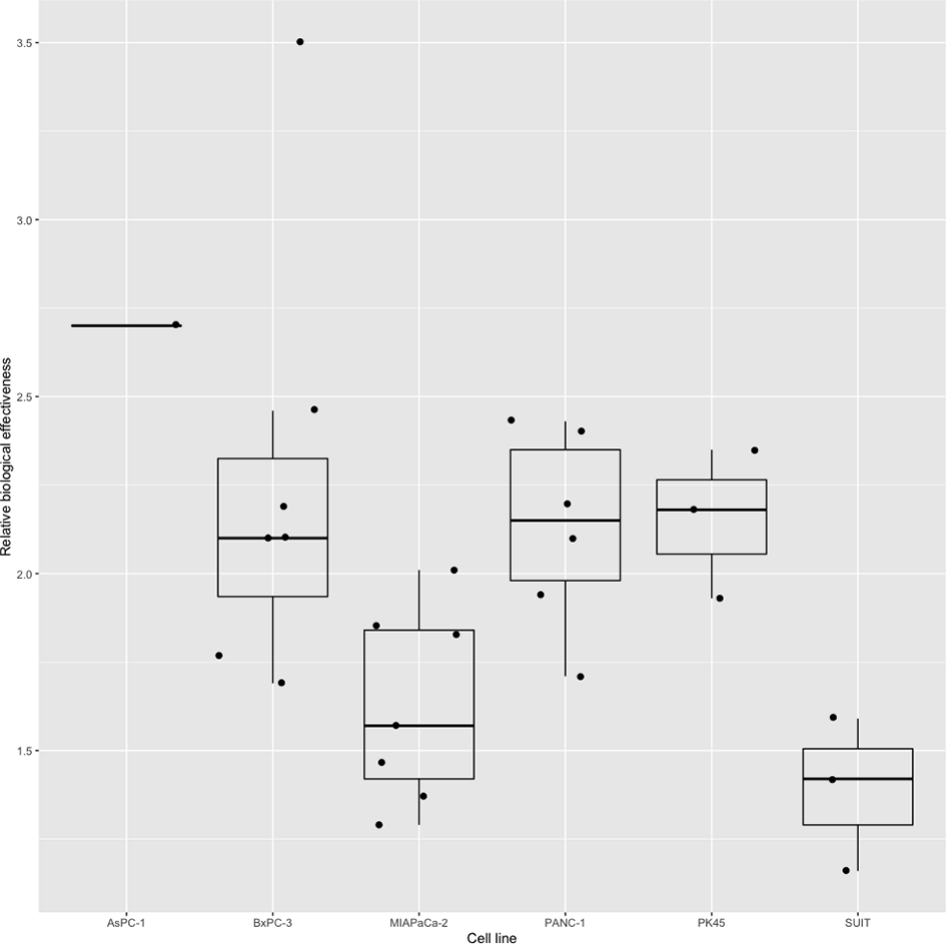
Boxplot representing the RBE value of carbon ion irradiation for the six human pancreatic cell lines.

Four studies (15, 18) (20, 34) used a linear-quadratic model to determine RBE values; the remaining 16 did not declare the model used. Nine studies using six different human pancreatic cancer cell lines in total reported RBE values for carbon ion irradiation, ranging from 1.29 to 4.5 (**Figure 3**). Two of these studies (14, 26) reported RBE values were higher for CSCs than non-CSCs for the same cell line. Matsui and colleagues (25) used three pancreatic cancer cell lines each irradiated with three different LETs and demonstrated that RBE values increased with increasing LET. Schlaich and colleagues (27) calculated RBE values for carbon ion irradiation alone (2.4 ± 0.4) and in combination with CPT (2.2 ± 0.2). Two studies (15, 28) reported the RBE values for proton irradiation, which varied from 0.6 to 2.1 depending on the pancreatic cell line used. Details are shown in **Table 2**.

**Table 2:** RBE values comparing carbon ion/proton to photon irradiation effectiveness in pancreatic cancer cell lines.

Author, year	RBE Model	RBE values of different cell lines
Oonishi, K. 2012 (14)	NR	MIA PaCa-2 (Unsorted): 1.85; BxPc-3 (Unsorted): 2.10; MIA PaCa-2 (CD44^+^/CD24^+^): 2.01; MIA PaCa-2 (CD44^-^/CD24^-^): 1.47; BxPc-3 (CD44^+^/CD24^+^): 2.19
Hartmann, L. 2020 (18)	L-Q Model	PDA30364/OVA: 3.23-10.0
Sai, S. 2015 (26)	NR	PANC1 (Unsorted): 1.71; PK45 (Unsorted): 2.18; PANC1 (CD44^+^/ESA^+^): 2.43; PANC1 (CD44^-^/ESA^-^): 1.94; PK45 (CD44^+^/ESA^+^): 2.35; PK45 (CD44^-^/ESA^-^): 1.93
Schlaich, F. 2013 (27)	NR	PANC-1(no drug): 2.4 ± 0.4; PANC-1(CPT): 2.2 ± 0.2
El Shafie, R. A. 2013 (20)	L-Q Model	Ranged from 1.5-4.5 depending on cell line and survival level
Matsui, Y. 2004 (25)	NR	MIAPaCa-2 (Carbon ion irradiation 13/50/80KeV/μm): 1.29/1.57/1.83; SUIT (Carbon ion irradiation 13/50/80KeV/μm): 1.16/1.42/1.59; BxPC-3 (Carbon ion irradiation 13/50/80KeV/μm): 1.77/1.69/2.46
Brero, F. 2020 (19)	NR	BxPC3: 3.5
Fujita, M. 2012 (23)	NR	PANC-1: 2.2
Lee, S. H. 2021 (34)	L-Q Model	MIAPaCa-2: 1.38*
Fujinaga, H. 2019 (28)	NR	MIAPaCa-2: 1.3
Görte, J. 2020 (15)	L-Q Model	MIAPaCa-2: 1.2; Capan-1: 1.2; Panc-1: 1.7; BxPC-3: 0.6; Patu8902: 1.4; Colo357: 2.1

CPT, camptothecin; L-Q Model, linear quadratic model; NR, not reported; RBE, relative biological effectiveness.

*at the center of the target region (=120mm).

### Clonogenic Survival

We calculated the SF for carbon ion, proton, and photon irradiation from radiation survival curves. The mean SF of 2 Gy (SF2) of carbon ion, proton, and photon irradiation was 0.18 ± 0.11, 0.48 ± 0.11, and 0.57 ± 0.13, respectively. The SF values associated with different radiation doses are shown in **Table 3**. Three studies (18, 20, 27) demonstrated that carbon ion irradiation had an enhanced efficacy in the suppression of clonogenic survival compared with standard photon irradiation in human pancreatic cell lines and pancreatic adenocarcinoma cell lines from transgenic mice. The presence of Olaparib (Ola, a PARP inhibitor), MNPs, or Hyp can enhance this cytotoxic effect. The SF for cancer stem cell-like CD44^+^/CD24^+^ or CD44^+^/ESA^+^ cells was found to be significantly higher than non-cancer stem-like CD44^–^/CD24^–^ or CD44^–^/ESA^–^ cells after treatment with either carbon ion or photon irradiation. The number of tumor spheroids was significantly lower in carbon ion-irradiated CSCs than in those that were irradiated with X-rays (14, 26). Matsui and colleagues (25) concluded that the survival rates of pancreatic cell lines after carbon ion irradiation were correlated with LET level and RBE values. Görte and colleagues (15) revealed that proton irradiation is associated with a greater reduction in PDAC tumoroid growth than X-ray irradiation.

**Table 3:** The SF values of pancreatic cancer cells irradiated by carbon ion, proton, and photon.

	Carbon ion irradiation	Proton irradiation	Photon irradiation
SF1	0.43 ± 0.15	0.70 ± 0.08	0.76 ± 0.09
SF2	0.18 ± 0.11	0.48 ± 0.11	0.57 ± 0.13
SF3	0.05 [0.03, 0.13]	0.33 ± 0.11	0.38 ± 0.15
SF4	0.04 ± 0.04	0.23 ± 0.11	0.22 [0.14,0.34]
SF5	0.02 ± 0.02	0.13 ± 0.08	0.13 [0.06,0.20]
SF6	0.01 [0.0008, 0.02]	0.05 [0.04,0.10]	0.08 [0.03,0.13]
SF7	–	0.02 [0.02,0.02]	0.04 [0.008,0.11]
SF8	–	0.008 [0.008,0.008]	0.03 [0.02,0.10]

SF, surviving fraction.

Data are mean ± SD or median (interquartile range).

### The Effects of DDR

Overall, ten studies investigated the DDR effects of carbon ion/proton irradiation on pancreatic cancer cells. Two of these (18, 25) reported that carbon ion irradiation induced more G2/M arrest compared with photon irradiation and that the intensity of G2/M arrest was stronger with an increased LET level. Combined GEM and carbon ion irradiation increased the expression of senescence-related genes such as *P21*, *P16*, and *P27* compared with carbon ion irradiation alone. The presence of Ola enhanced G2/M phase arrest and reduced the level of histone H3 phosphorylation induced by photon or carbon ion/proton irradiation. One study (29) confirmed proton irradiation can induce significant cell cycle arrest in a dose-dependent manner in both radiosensitive and radioresistant pancreatic cell lines.

Compared with unirradiated controls, Brero and colleagues (19) found that carbon ion irradiation alone increased the formation of γH2AX and 53BP1 foci, which were validated markers of DNA DSBs. Oonishi and colleagues (14) reported on the expression of *γH2AX* in human pancreatic CSCs after irradiation. The number of γH2AX foci in CD44^–^/CD24^–^ cells were higher than that of CD44^+^/CD24^+^ cells after irradiation with either carbon ion or X-rays, and the number of γH2AX foci in CD44^+^/CD24^+^ cells irradiated with carbon ion persisted significantly longer compared with those irradiated with X-rays. Meanwhile, the authors discovered that carbon ion irradiation alone can also strongly increase in the size of γH2AX foci (clustered DNA damage). Sai and colleagues (26) confirmed that combined therapy with carbon ion irradiation and GEM significantly increased the expression of DNA damage and repair-related genes such as *ARTEMIS*, *RAD51*, *TP53BP1*, and *BRAC1*. Compared with unirradiated cells, the expression of *γH2AX* was significantly increased in Capan-1, Panc-1, and MIA PaCa-2 cells after proton irradiation. Lee and colleagues (32) demonstrated that the level of the DNA repair protein RAD51 was dose-dependently decreased in Capan-1cells, but not in Panc-1 cells. Two studies (24, 31) reported the expression of *γH2AX* was increased and prolonged following carbon ion/proton irradiation in the presence of Ola.

Sai and colleagues (26) reported that carbon ion irradiation combined with GEM increased the level of apoptosis in PK45 cells, and the apoptosis-related gene expressions such as *Bax*, *cytochrome c*, and *Bcl2* were significantly elevated with combined carbon ion irradiation/GEM and with GEM alone. One study (25) demonstrated that carbon ion irradiation induced mitotic death, rather than apoptotic death, in BxPC-3, MIAPaCa-2, and SUIT2 cells. The effects of proton irradiation on apoptosis varied according to cell type: in Capan-1 cells, proton irradiation increased the expression of cleaved PARP, which is known as a marker for apoptotic cell death (32). Proton irradiation alone increased the levels of survivin in MIA PaCa-2 cells with little apoptosis. However, combined proton and GEM therapy induced robust apoptosis with a concomitant reduction in survivin and XIAP (29). In radiation-resistant PANC-1cells, two studies (29, 32) showed that proton irradiation increased survivin levels with little apoptosis, even in combination with GEM. Details are presented in **Table 4**.

**Table 4:** DDR of pancreatic cancer cells after carbon ion/proton irradiation.

Author, year	Treatment	Outcome assessed	Finds
Oonishi, K. 2012 (14)	Carbon ion irradiation	DNA repair	The number of γH2AX foci in CD44-/CD24- cells was higher than that of CD44+/CD24+ cells; ↑ the size of γH2AX foci
Hartmann, L. 2020 (18)	Carbon ion irradiation	Cell cycle checkpoints	G2/M arrest by a transient and dose-dependent manner
Sai, S. 2015 (26)	Carbon ion irradiation + GEM	Cell cycle checkpoints	↑ senescence-related genes such as *p21*, *p16* and *p27* expression
		DNA repair	↑ the number and the size of γH2AX foci; ↑ DNA damage and repair-related genes such as *ARTEMIS*, *Rad51*, *TP53BP1*, *BRAC1*
		Apoptosis	Induce the apoptosis of CSCs and non-CSCs; ↑ apoptosis-related gene expressions such as *Bax*, *cytochrome c* and *Bcl2*
Hirai, T. 2012 (24)	Carbon ion irradiation + Ola	Cell cycle checkpoints	G2/M arrest; ↓ phosphorylated histone H3
		DNA repair	↑ levels of γ-H2AX
Matsui, Y. 2004 (25)	Carbon ion irradiation	Cell cycle checkpoints	G2/M arrest
		Apoptosis	Irradiation induced mitotic death rather than apoptotic death
Brero, F. 2020 (19)	Carbon ion irradiation	DNA repair	↑ the number of DSBs markers (γH2AX and 53BP1)
Lee, Min-Gu 2019 (32)	Proton irradiation	Cell cycle checkpoints	↑ p21 protein expression; ↓ Phosphorylated STAT3
		DNA repair	↑ phosphorylation of H2A.X; ↓ the expression of RAD51 protein in Capan‐1 cells
		Apoptosis	↑ survivin gene and protein expression in Panc-1 cells; ↑ cleaved PARP in Capan-1 cells
Liubavičiūtė, A. 2015 (30)	Proton irradiation	Cell cycle checkpoints	Temporary G1/0 cell cycle arrest
		DNA repair	The cells expressing the γH2AX at 1 h, 3 h, 6 h, 24 h, 48 h, and 72 h after Proton irradiation was 97%, 93.43%, 83.47%, 62.3%, 23.1%, and 3.78%, respectively
		Apoptosis	The percentage of apoptotic cells 24 h after irradiation was 45%; after 48 h, 60%, and after 72 h, 79%
Galloway, N. R. 2009 (29)	Proton irradiation + GEM	Cell cycle checkpoints	G2/M arrest*; G0/G1 arrest†
		Apoptosis	Robust apoptotic induction; ↓ survivin and XIAP in the MIA PaCa-2 cells
Hirai, T. 2016 (31)	Proton irradiation + Ola	Cell cycle checkpoints	↑ p-p53; S phase arrest with a subsequent G2/M arrest
		DNA repair	↑ the number of γH2AX foci/nucleus

CSC, cancer stem-like cells; DDR, DNA damage response; DSB, double-strand break; GEM, gemcitabine; Ola, Olaparib; PARP, poly (ADP-ribose) polymerase; XIAP, X-linked inhibitor of apoptosis protein.

*sequential treatments that used proton irradiation as the first modality in the treatment regimen; †GEM as the first modality; ↑: increase; ↓: reduction.

### Migration and Invasion Ability

There were five publications (13, 21–23, 26) involved migration, invasion, or the expression of related genes (**Table 5**). Fujita and colleagues (13, 22, 23) conducted a series of studies and demonstrated carbon ion irradiation (2Gy) could repress the migration of AsPC, BxPC-3, and MIAPaCa-2 cells, diminish MIAPaCa-2 cell invasiveness *via* prevented the activity of Rac 1 and RhoA, and reduce the invasiveness of AsPC, BxPC-3 *via* inhibited the activity of Rac 1 through Ub-mediated proteasomal degradation. In contrast, 2Gy carbon ion irradiation promoted the invasion of PANC-1 cells by activating of plasmin and urokinase-type plasminogen activator, and nitric oxide also played an important role in this process *via* activation of the PI3K-AKT and RhoA pathways. Facoetti and colleagues (21) reported the migration of AsPC-1 cells is regulated by components released by normal fibroblasts and tumor cells through morphological analysis. Sai and colleagues (26) demonstrated carbon ion or photon irradiation alone and/or in combination with GEM in PK 45 cells could enhance the level of tumor invasion-related genes like *MMP-2*, *MMP-9*, *E-cadherin*, and *β-catenin*.

**Table 5:** Migration and invasion of pancreatic cancer cells after carbon ion irradiation.

Author, year	Migration and invasion ability
Sai, S. 2015 (26)	Tumor invasion-related genes like *MMP2*, *MMP9*, *E-cadherin* and *β-catenin* were increased by either carbon ion or X-ray irradiation alone and/or in combination with GEM.
Fujita, M. 2015 (13)	The Ub-proteasome-mediated degradation of Rac1 and RhoA is a mechanism underlying the suppression of MIAPaCa-2 cell motility by carbon ion irradiation.
Fujita, M. 2012 (23)	Carbon ion irradiation is effective in suppressing the invasive potential of MIAPaCa-2, BxPC-3 and AsPC-1cells; Carbon ion irradiation increased the invasiveness of PANC-1 through the activation of plasmin and urokinase-type plasminogen activator.
Facoetti, A. 2018 (21)	The migratory behavior of Aspc-1 cells is modulated by factors released by normal fibroblasts and tumor cells, and this is in turn modulated by both the radiation dose and the radiation quality.
Fujita, M. 2014 (22)	Nitric oxide increases the invasion of PANC-1 cells *via* activation of the PI3K–AKT and RhoA pathways after carbon ion irradiation.

GEM, gemcitabine.

### SER Evaluating the Therapeutic Effect of Combination Therapy

**Figure 4:**
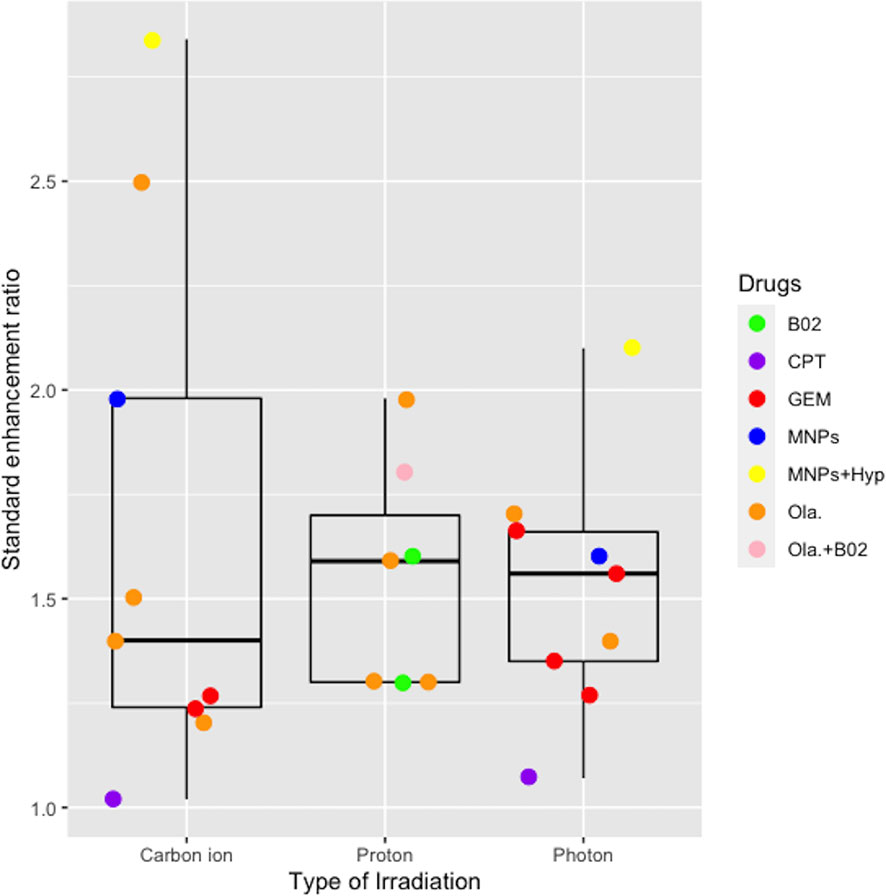
Boxplot representing the SER value of combined effects of chemotherapy or target therapy with the carbon ion, proton, and photon irradiation. B02, a RAD51 inhibitor; CPT, camptothecin; GEM, gemcitabine; MNPs, magnetic nanoparticles; Hyp, hyperthermia; Ola, Olaparib.

Six studies reported on the therapeutic effects of combination therapy in five different cell lines exposed to carbon ion/proton irradiation (**Table 6**). SER values depended on the type of irradiation, ranging from 1.02–2.81 for carbon ion, 1.30–1.98 for proton, and 1.07–2.10 for photon irradiation, respectively. Two publications (24, 31) reported the SER associated with low versus high LET radiation levels, confirming that the high LET levels had an enhanced effect. Brero and colleagues (19) demonstrated that combination of MNPs and Hyp substantially enhanced the effects of irradiation, irrespective of the type of irradiation used. CPT had a slight enhancing effect on both carbon ion and photon irradiation (27). Wera and colleagues (33) revealed that the combination of Ola and B02 further sensitized KP4 cells, but not PANC-1 cells, to proton irradiation. Taken together, the pooled analysis indicated that combination therapies enhanced the therapeutic effects of pancreatic cell lines with a mean SER of 1.66 ± 0.63 for carbon ion, 1.55 ± 0.27 for proton, and 1.52 ± 0.30 for photon irradiation (**Figure 4**). However, no significant differences compared with photon irradiation were observed for carbon ion or proton irradiation (*P* > 0.05).

**Table 6:** SER values determined from combination therapy.

Author, year	Combination therapy (Dose)	Cell line	SER		
			Carbon ion irradiation	Proton irradiation	Photon irradiation
Hirai, T. 2012 (24)	Ola 1 μM	MIA PaCa-2	1.2; 1.4*	–	1.4
	Ola 5 μM	MIA PaCa-2	1.5; 2.5*	–	1.7
Schlaich, F. 2013 (27)^†^	CPT 25 nM	PANC-1	1.02	–	1.07
El Shafie, R. A. 2013 (20)	GEM 10 nM	AsPC-1	1.24	–	1.27
	GEM 50 nM	AsPC-1	1.27	–	1.66
	GEM 10 nM	PANC-1	–	–	1.56
	GEM 50 nM	PANC-1	–	–	1.35
Brero, F. 2020 (19)^†^	MNPs	BxPC-3	1.98	–	1.60
	MNPs+Hyp	BxPC-3	2.84	–	2.10
Wera, Anne-Catherine 2019 (33)	Ola 0.5μM	KP4	–	1.3	–
	B02 10μM	KP4	–	1.3	–
	Ola 0.5μM + B02 0.5μM	KP4	–	1.8	–
	Ola 0.5μM	PANC-1	–	1.3	–
	B02 10μM	PANC-1	–	1.6	–
Hirai, T. 2016 (31)	Ola 5 μM	MIA PaCa-2	–	1.59; 1.98^‡^	–

CPT, camptothecin; GEM, gemcitabine; Hyp, hyperthermia; MNP, magnetic nanoparticles; Ola, Olaparib; SER, standard enhancement ratio.

*Cells treated with LET 13 keV/μm and LET 70keV/μm, respectively; ^†^Data extracted from cell survival curves; ^‡^Cells treated at entrance region (ER) and at Bragg peak (BP), respectively.

## Discussion

This SR aimed to evaluate the radiobiological effects of charged particle irradiation on pancreatic cancer cell lines, including cell survival, DDR, migration, and invasion ability. In total, 20 *in vitro* studies, including 13 on carbon ion irradiation and seven on proton irradiation, were included. Our results revealed that carbon ion irradiation had superior radiobiological effects on pancreatic cancer cells when compared with photon irradiation. The lack of a direct comparison between proton and photon irradiation across the studies included here prevents any similar conclusions for proton irradiation. Both carbon ion irradiation and proton irradiation confer similar sensitization effects in comparison with photon irradiation when combined with chemotherapy or targeted therapy.

RBE is a complex quantity that depends on physical parameters, such as particle type and energy, LET and dose, as well as biological parameters, including tissue/cell type, cell cycle phase, oxygen level, and endpoint (38). Acceptable RBE values are generally considered to be approximately 1.1 for protons and 2.5– 3 for carbon ions (39), which is generally in line with the reported RBE values included in this SR. However, Gorte and colleagues (15) showed that the RBE value associated with proton irradiation in pancreatic cancer cell lines varied from 0.6 in BxPC-3 cells to 2.1 in Colo357 cells. The considerable uncertainty and great variability in the determination of absolute RBE values have long been recognized (40, 41). Furthermore, some hold the perspective that the RBE is a clinically relevant parameter, the clinical RBE involves a medical decision based on empirical data and clinical information. So it should be determined under *in vivo* growth conditions such as animal models, which enable simulation of clinical treatments, especially in late tissue vascular and fibrotic complications (42). Further research is needed to obtain accurate RBE values for carbon ion/proton irradiation, and thus to understand the mechanisms underpinning the enhanced effectiveness of high LET irradiations and apply them in a clinical setting.

Our SR revealed greater growth inhibition with carbon ion/proton irradiation compared with photon irradiation, which is attributed to the capacity to cause damage to cellular macromolecules, particularly DNA. Radiation-induced DNA damage is classified as direct (ionizing radiation interacts directly with the DNA) and indirect [mediated through reactive oxygen species (ROS)]. With increased LET, and thus increasing ionization density, the proportion of direct damage, the complexity of the DNA damage pattern, and the release of DNA fragments are also increased, and the repairability of the managed site is decreased (43, 44). This is consistent with the phenomenon observed among the studies we included in this SR, which found a strong increase of clustered DNA damage in carbon ion irradiated cells, which are difficult to repair (14, 26). Meanwhile, the less reliance on indirect DNA damage mediated by ROS makes charged particles more effective in treating pancreatic cancers, which is very hypoxic. The complexity of the DNA damage and the lower efficiency of DNA repair leads to increased effectiveness and tolerable toxicity levels with CIRT/PRT in the treatment of cancers (7, 45). The energy absorption of photons and carbon ions/protons are fundamentally different: photons lose their energy exponentially, with higher values at the physical entry point and lower values in deeper tissues (46), while carbon ions/protons deposit most of their energy at the end of the physical range of the particles and reaches the peak, forming the so-called Bragg-peak. After the Bragg-peak, the delivered dose drops rapidly with increased depth. This enables charged particles to target tumors more precisely while reducing the dose and irradiated portion of normal tissue. Compared with IMRT, both intensity-modulated carbon ion therapy and intensity-modulated proton therapy can reduce the OARs integral dose substantially in paraspinal sarcomas, locally recurrent nasopharyngeal carcinoma, and other cancer types in clinical settings (47, 48).

Cell cycle arrest can be induced in the G1/0 or S phase, in which DNA repair occurs *via* the non-homologous end joining (NHEJ) pathway, or the G2 phase, in which repair occurs *via* the homologous recombination (HR) pathway (49). Previous studies have demonstrated that HR is relatively low after photon irradiation in PDAC cells (50). Our results showed that, compared with photon, carbon ion irradiation induced greater G2/M arrest and longer-lasting expression of *γH2AX* as well as an increase in γH2AX clusters, indicating a high level of DNA damage. These clustered DNA lesions are difficult to repair and may induce different signaling pathways compared with photon irradiation, such as DNA repair, type I interferon signaling, and cell cycle pathways (51, 52). Unrepaired or misrepaired damage can inhibit cell proliferation *via* checkpoints; cells may therefore stay in the G2/M phase, undergoing genomic instability or cell death by several mechanisms, including apoptosis, mitotic catastrophe, or senescence. This is consistent with the results in a study by Gerelchuluun and colleagues, who used Chinese hamster cell lines (ovary AA8 and lung fibroblast V79), which demonstrated that the primary DNA repair pathway after both proton and carbon ion radiation is NHEJ, but activity in the HR pathway is greater with carbon ion (53).

In PDAC, mutations in four genes predominate: *KRAS*, *TP53*, *SMAD4*, and *CDKN2A*, each of which is mutated in >50% of different but mostly overlapping patients (54), but a mutation in the *KRAS* oncogene is present in >90% of cases (55), which may lead to resistance to photon irradiation. One included study (32) demonstrated that increased *P21* expression after proton irradiation is correlated with inhibition of STAT3 phosphorylation, indicating that proton irradiation is likely to induce cell cycle arrest and/or cell death *via* the regulation of STAT3 signaling, irrespective of radiosensitivity. Correspondingly, another study (25) demonstrated carbon ion irradiation arrested cell cycle progression in an LET-dependent manner, independent of gene mutation status. This finding is supported in other research (56, 57). We therefore hypothesize that charged particle irradiation with high-LET may induce widely and densely distributed, irreparable DNA breaks and subsequent cell death.

The mammalian Rho family of GTPases consists of 22 members. Rho functions as a molecular switch in cellular processes such as cell morphogenesis, adhesion, migration, and cell cycle progression including cytokinesis (58). Rho-associated coiled-coil forming kinase (ROCK) is a serine/threonine kinase that can phosphorylate a variety of substrates and is one of the major effectors for Rho GTPases. During migration, tumor cells display a great variety of morphological changes; the modalities of single-cell migration can be subdivided into amoeboid and mesenchymal (59). Cells in the amoeboid mode are regulated by ROCK, while cells in the mesenchymal mode depend on the proteolytic activity of matrix metalloproteinases (MMPs), which permit penetration of the extracellular matrix (ECM). As shown by the studies included in this SR, carbon ion irradiation can suppress the invasive potential of pancreatic cells *via* Rho/ROCK signaling, except in PANC-1 cells. However, the combination of serine protease inhibitors (SerPI) and ROCK inhibitors may suppress the invasion of PANC-1 cells. Aspc-1 cells were preponderant in mesenchymal phenotypes following carbon ion irradiation; this effect could be inhibited by MMP inhibitors (23). Studies on the mechanism underlying the effect of carbon ion/proton irradiation on invasion and migration of pancreatic cancer cells remains limited, and mainly focus on the Rho/ROCK signaling pathway, which is known to be associated with metastasis (60, 61). More in-depth research on this topic is required; moreover, carbon ion/proton irradiation combined with MMP or ROCK inhibitors may suppress invasion and migration.

All the included studies demonstrated the enhanced therapeutic effect of combined therapies. Similarly, Waissi and colleagues (51) confirmed that a combination of GEM, Ola, and proton irradiation can significantly improve local control of pancreatic cancer *in vivo*. A phase I trial (62) of PARPi in combination with GEM and photon radiotherapy in LAPC has shown that this regimen is safe and tolerable, with a median OS of 15 months. No clinical studies on particle therapy with PARPi have been published until now, but DNA damage induced by charged particles is more complicated than that induced by photons, and this damage is mainly repaired by base excision repair—in which PARP plays a significant and predominant role (63, 64). Thus, charged particle therapy in combination with PARPi may benefit pancreatic cancer patients, and further clinical studies should therefore be prioritized. One clinical trial (9) demonstrated that the 2-year OS in people with LAPC who received CIRT and concurrent GEM therapy was 35%; another phase I/II study (7) of PRT with concurrent GEM therapy for LAPC revealed a 1-year OS of 76.8%, this supports the theory that a combination of GEM chemotherapy and irradiation could benefit people with LAPC. The presence of MNPs and Hyp have been shown to have the strongest sensitization effect on both carbon ion and photon irradiation in this SR; similarly, Ma and colleagues (65) suggested that the combination of MNPs and Hyp has a similar effect on human nasopharyngeal and lung cancer cells, which may be related to their inhibition of DNA repair and induction of apoptosis.

As far as we know, this is the first SR of *in vitro* studies to evaluate the radiobiological responses of charged particle irradiation on pancreatic cancer cell lines. First, we systematically reviewed published studies on the radiobiological effects of carbon ion/proton irradiation on pancreatic cancer cell lines, including RBE, cell survival, DDR, invasion, migration, and the combined effects of chemotherapy and targeted therapy; this provided a comprehensive overview and avenues for further study. Second, we conducted a radiation-related risk of bias assessment tool for *in vitro* studies after reference to the Cochrane risk-of-bias tool (66), and the SYstematic Review Center for Laboratory animal Experimentation (SYRCEL) risk-of-bias tool for animal studies (67). This provides a reference for future radiation-related *in vitro* SRs. However, there are several limitations to this SR. First, searching only English databases may result in certain language bias. Second, the limited number of publications evaluating radiobiological responses in pancreatic cancer, and the heterogeneity of outcome assessment tools, mean that the strength of our findings may also be limited. However, we retrieved and obtained all available data, together representing the latest evidence for carbon ion/proton irradiation on pancreatic cancer *in vitro*; with the increasing application of carbon ion/proton irradiation worldwide, this review may provide at least a starting point to improve guidance for clinical practice. Third, the endpoints we selected are common and important indicators of the radiobiological effects of charged particle irradiation; however, other endpoints are equally important, for example the production of ROSs, which could provide insights into indirect DNA damage, or the expression of immunomodulatory molecules, which may help improve understanding of the effects of radioimmunotherapy. However, these other endpoints have been only rarely reported now; updates to this review may therefore be warranted in future.

## Conclusions

Current *in vitro* evidence demonstrates that, compared with photon irradiation, carbon ion irradiation offers superior radiobiological effects in the treatment of pancreatic cancer. Mechanistically, high-LET irradiation may induce complex DNA damage and ultimately promote genomic instability and cell death. Carbon ion irradiation can also effectively suppress the invasion and migration of most pancreatic cancer cell lines, but the mechanism is not well understood. The lack of direct comparisons between proton and photon irradiation prevents similar conclusions from being reached for this therapeutic option. Both carbon ion irradiation and proton irradiation confer similar sensitization effects in comparison with photon irradiation when combined with chemotherapy or targeted therapy.

## Data Availability Statement

The original contributions presented in the study are included in the article/**Supplementary Material**. Further inquiries can be directed to the corresponding author.

## Author Contributions

Guarantor of the article is XW. XW conceived and designed this article. JC, ZLi, YO, and QZ conducted literature searches, selected studies, and assessed the risk of bias. HL, YW, and RL extracted data. DW, MD, and ZLiu carried out analyses, interpreted results. DW, SS, KY, and JT drafted the manuscript. The lead and corresponding author (XW) affirm that the manuscript is an honest, accurate, and transparent account of the study being reported; that no important aspects of the study have been omitted. All authors contributed to the article and approved the submitted version.

## Funding

This study was supported by Science and Technology Plan Project of Chengguan District of Lanzhou (No.2020-2-2-5); Talent innovation and venture project of Lanzhou city (No. 2017-RC-23); Talent innovation and venture project of Lanzhou city (No. 2020-RC-113); Key R&D Program of Science and Technology Department of Gansu Province (No. 20YF8FA116); The authorized project of Lanzhou KejinTaiji Corporation, Ltd (No. BMP-B-02-002). The funder was not involved in the study design, collection, analysis, interpretation of data, the writing of this article or the decision to submit it for publication.

## Conflict of Interest

The authors declare that the research was conducted in the absence of any commercial or financial relationships that could be construed as a potential conflict of interest.

## Publisher’s Note

All claims expressed in this article are solely those of the authors and do not necessarily represent those of their affiliated organizations, or those of the publisher, the editors and the reviewers. Any product that may be evaluated in this article, or claim that may be made by its manufacturer, is not guaranteed or endorsed by the publisher.
