# Supplementary material for: Charged Particle Irradiation for Pancreatic Cancer: A Systematic Review of In Vitro Studies
Source: Front Oncol. 2022 Jan 4;11:775597. doi: 10.3389/fonc.2021.775597 (PMC8764177; doi:10.3389/fonc.2021.775597)
Supplement: Supplementary file 1 [file DataSheet_1.docx]

Supplementary Material

**Supplementary literature-search methods**

**Search Strategy in MEDLINE**

Ovid Technologies, Inc. Search limit to English language Database: Ovid MEDLINE(R) In-Process & Other Non-Indexed Citations and Ovid MEDLINE(R) <1946 to 2021 August 27 > Search Strategy:

1. exp Heavy Ion Radiotherapy/

2. exp Proton Therapy/

3. heavy ion radiotherapy.af.

4. heavy ion therapy.af.

5. heavy ion radiation therapy.af.

6. particle beam therapy.af.

7. carbon ion therapy.af.

8. carbon ion radiation therapy.af.

9. carbon ion radiotherapy.af.

10. carbon ion irradiation.af.

11. carbon ion radiotherapy.af.

12. proton therapy.af.

13. proton radiation.af.

14. proton irradiation.af.

15. exp Pancreatic Neoplasms/

16. pancreatic neoplasms.af.

17. pancreas cancer.af.

18. pancreatic cancer.af.

19. pancreatic adenocarcinoma.af.

20. pancreatic ductal adenocarcinama.af.

21. 1 or 2 or 3 or 4 or 5 or 6 or 7 or 8 or 9 or 10 or 11 or 12 or 13 or 14

22. 15 or 16 or 17 or 18 or 19 or 20

22. 21 and 22

**Search Strategy in EMBASE**

Ovid Technologies, Inc. Search limit to English language Database: EMBASE Classic + EMBASE <1947 to 2021 August 27 > Search Strategy:

1. exp ion therapy/

2. exp heavy ion radiation/

3. exp heavy ion/

4. exp proton therapy/

5. heavy ion therapy.af.

6. heavy ion radiotherapy.af.

7. heavy ion radiation therapy.af.

8. proton therapy.af.

9. proton radiation.af.

10. proton irradiation.af.

11. proton radiotherapy.af.

12. carbon ion therapy.af.

13. carbon ion radiation therapy.af.

14. carbon ion radiotherapy.af.

15. carbon ion irradiation.af.

16. exp pancreas cancer/

17. pancreas cancer.af.

18. pancreatic neoplasms.af.

19. pancreatic ductal adenocarcinama.af.

20. pancreatic adenocarcinoma.af.

21. pancreatic cancer.af.

22. 1 or 2 or 3 or 4 or 5 or 6 or 7 or 8 or 9 or 10 or 11 or 1 or 13 or 14 or 15

23. 16 or 17 or 18 or 19 or 20 or 21

24. 22 and 23

**Search Strategy in Web of Science**

WOS, BIOSIS, KJD, RSCI, SCIELO Time span = all years, search language = automatic

1.TS=(proton therapy OR proton radiation OR proton irradiation OR proton beam radiation OR proton beam irradiation OR proton radiotherapy)

2.TS=(particle therapy OR heavy ion radiation OR heavy ion irradiation OR heavy ion radiotherapy OR heavy ion therapy OR particle radiation OR particle beam irradiation OR particle radiotherapy OR particle beam radiation)

3.TS=(carbon ion therapy OR carbon ion radiation therapy OR carbon ion irradiation OR carbon ion radiotherapy)

4.TS=(pancreatic neoplasms OR pancreatic ductal adenocarcinoma OR pancreatic adenocarcinoma OR pancreatic cancer OR pancreas cancer)

5. #3 OR #2 OR #1

6. #5 AND #4

Table S1. Risk of bias scheme

|  |  | Yes | Partly | No | Risk unknown | Not applicable |
| --- | --- | --- | --- | --- | --- | --- |
| Selection bias | Was the method of cell counting described? | Yes | - | No | - | - |
| Performance bias | Were the details of irradiation described? | Initial energy, averaged LET, single dose | Described part of them | No | - | - |
|  | Was the exposure blinded? | Yes | - | No | Not reported | Not applicable |
|  | Was the implementation process of experiment reported? | Described every process involved | Described part of them | Not reported | - | - |
| Detection bias | Were the methods of measuring the results described? | Described every method involved | Described part of them | No | - | - |
| Attrition bias | Whether the data of experimental results were lost or whether the experiment was not repeated? | Yes | Lost data or did not repeat | No | - | - |
| Cell related bias | Were the culture conditions of cell reported? | Yes | - | Not reported | - | - |
|  | Was the cell origin and cell type used reported? | Yes | Cell origin or cell type reported | Not reported | - | - |
| Other bias | Was there industry sponsoring involved? | Yes | - | No | Not reported | - |
